# Prevalence of HIV infection in patients of a substance use treatment facility

**DOI:** 10.1192/j.eurpsy.2023.1384

**Published:** 2023-07-19

**Authors:** L. T. Baranskaya, E. Babushkina, V. Potapov

**Affiliations:** Psychiatry, Psychotherapy and Narcology, Ural State Medical University, Yekaterinburg, Russian Federation

## Abstract

**Introduction:**

According to domestic and foreign studies, in the last decade there has been an increase in the number of HIV-infected patients suffering from alcoholic disease. Under the influence of alcohol, the risk of infection and transmission of HIV infection increases, the course of the disease worsens. There is a syndemia between alcohol abuse and HIV infection. This requires new approaches to the tactics of managing patients of a narcological hospital with HIV infection.

**Objectives:**

To identify the prevalence of HIV infection in patients of the substance use treatment facility suffering from alcohol disease of the second stage.

**Methods:**

The authors analyzed 446 medical histories of patients suffering from alcoholic disease who underwent inpatient treatment in 2009-2021. For data processing, a statistical method for calculating relative indicators used.

**Results:**

The study showed that a significant proportion of patients with HIV infection were patients of working age 40-59 years. At the same time, since 2009, there has been an increase in HIV-infected patients from the total number of hospital patients. So, if in 2009 they were 1.2%, then in 2021 they were already 7.2%. Most HIV-infected patients of the narcological hospital are single or divorced. The study notes that abuse is common among patients with HIV infection as a means of combating depression. According to 2019 data, 39.0% of hospital patients suffer from depression. The authors show that alcohol affects the increase in risky forms of sexual relations, or risky situations with unprotected sex. In addition, with alcohol abuse, the viral load increases, and therefore the likelihood of transmitting HIV infection to a partner with unprotected sex increases.

**Conclusions:**

Alcohol abuse is a causative factor in HIV infection. Early detection of people with harmful alcohol consumption and referral to a psychiatrist will increase adherence to antiretroviral therapy, as well as reduce the rate of HIV infection

**Disclosure of Interest:**

None Declared

